# Oxidant-NO dependent gene regulation in dogs with type I diabetes: impact on cardiac function and metabolism

**DOI:** 10.1186/1475-2840-9-43

**Published:** 2010-08-24

**Authors:** Caroline Ojaimi, Shintaro Kinugawa, Fabio A Recchia, Thomas H Hintze

**Affiliations:** 1 Department of Physiology, New York Medical College, Valhalla, NY 10595, USA

## Abstract

**Background:**

The mechanisms responsible for the cardiovascular mortality in type I diabetes (DM) have not been defined completely. We have shown in conscious dogs with DM that: *1*) baseline coronary blood flow (CBF) was significantly decreased, *2*) endothelium-dependent (ACh) coronary vasodilation was impaired, and *3*) reflex cholinergic NO-dependent coronary vasodilation was selectively depressed. The most likely mechanism responsible for the depressed reflex cholinergic NO-dependent coronary vasodilation was the decreased bioactivity of NO from the vascular endothelium. The goal of this study was to investigate changes in cardiac gene expression in a canine model of alloxan-induced type 1 diabetes.

**Methods:**

Mongrel dogs were chronically instrumented and the dogs were divided into two groups: one normal and the other diabetic. In the diabetic group, the dogs were injected with alloxan monohydrate (40-60 mg/kg iv) over 1 min. The global changes in cardiac gene expression in dogs with alloxan-induced diabetes were studied using Affymetrix Canine Array. Cardiac RNA was extracted from the control and DM (n = 4).

**Results:**

The array data revealed that 797 genes were differentially expressed (P < 0.01; fold change of at least ±2). 150 genes were expressed at significantly greater levels in diabetic dogs and 647 were significantly reduced. There was no change in eNOS mRNA. There was up regulation of some components of the NADPH oxidase subunits (gp91 by 2.2 fold, P < 0.03), and down-regulation of SOD1 (3 fold, P < 0.001) and decrease (4 - 40 fold) in a large number of genes encoding mitochondrial enzymes. In addition, there was down-regulation of Ca^2+ ^cycling genes (ryanodine receptor; SERCA2 Calcium ATPase), structural proteins (actin alpha). Of particular interests are genes involved in glutathione metabolism (glutathione peroxidase 1, glutathione reductase and glutathione S-transferase), which were markedly down regulated.

**Conclusion:**

our findings suggest that type I diabetes might have a direct effect on the heart by impairing NO bioavailability through oxidative stress and perhaps lipid peroxidases.

## Background

Diabetes mellitus (DM) is a multifactorial disease which is characterized by hyperglycemia, lipoprotein abnormalities [1] and altered intermediary metabolism of major food substrates [2]. In addition, the generation of free radicals often worsens the complications of DM such as hypertension, atherosclerosis and microcirculatory disorders [3,4]. Changes in lipid levels and consequent disorders of lipid metabolism and stress have been observed in DM [5]. Due to altered intermediary metabolism in DM, a feature present in both type 1 and type 2 DM, patients with type 2 DM, even in the absence of clinical cardiovascular disease, have a reduced maximal oxygen consumption compared with non-diabetic people [6]. On the other hand, several studies have reported that the basal metabolic rate is increased in diabetic states [7,8]. This increase is a reflection of the increased metabolic demands in DM.

Diabetes mellitus is usually associated with coronary artery disease. Since the discovery of nitric oxide (NO), a number of studies have revealed impaired endothelium-dependent vasorelaxation in diabetic animals [9,10]. Our previous study [11] has indicated that reflex, NO-dependent coronary vasodilation is depressed in conscious dogs after the development of alloxan-induced diabetes, suggesting a reduced ability of coronary blood vessels to produce NO. Furthermore, in another study [12], we have shown that: 1) kallikrein causes a decrease in O_2 _consumption in cardiac muscle from normal dogs, which is mediated by kinin and NO mechanisms; and *2*) the modulation of O_2 _consumption by endogenous NO is depressed in cardiac muscle from diabetic dogs, whereas the modulation of O_2 _consumption by exogenous NO is still preserved. The mechanism responsible for the depressed modulation of O_2 _consumption by endogenous NO is likely because of the decreased release of NO from the vascular endothelium rather than mitochondrial dysfunction. Various studies have shown that there is also evidence for mitochondrial dysfunction in the heart from the diabetic rat and mouse [13-16]. In addition, another study by Linke et al [17] showed significant changes in the myocardial substrate (glucose) utilization in dogs only in the late stage of diabetes at a time when myocardial NO production is already decreased. DNA microarrays have been used in every aspect of biological and medical research, including obesity and type 2 diabetes mellitus (T2D) in human and various small animal models [18]. Therefore, we sought to investigate cardiac gene expression changes in a canine model of alloxan-induced type 1 diabetes. We subsequently, compared and contrasted these changes to the cardiac functional and metabolic effects that we previously observed.

## Methods

### Surgical instrumentation and *in vivo *protocol

The protocols were approved by the Institutional Animal Care and Use Committee of New York Medical College and conform to the guiding principles for the use and care of laboratory animals of the National Institutes of Health and the American Physiological Society. The loss of body weight in the diabetic dogs was specifically discussed at the meeting of the Institutional Animal Care and Use Committee. Dogs were weighed every other day.

#### Surgical Preparation

Male Mongrel dogs (24.5-29 kg body wt, *n *= 8) were premedicated with acepromazine (0.3 mg/kg im) and anesthetized with pentobarbital sodium (25 mg/kg iv). A thoracotomy was performed, and instruments were implanted for measurement of pressure, flow, and blood sampling as described previously [11].

#### Induction of Diabetes in Conscious Dogs

The protocol used for these groups of control and diabetic dogs was the same as reported in our previous studies [11,12,17]. Mongrel dogs were chronically instrumented for measurements of systemic hemodynamics. The control hemodynamics were recorded 10-14 days after the surgery. After the control recording, the dogs were divided into two groups: one normal (*n *= 4) and the other diabetic (*n *= 4). In the diabetic group, the dogs were injected with alloxan monohydrate (40-60 mg/kg iv) over 1 min. Alloxan was prepared as a 5% solution in citrate buffer (pH 4-4.5). Blood gases and plasma glucose were measured on *days 3 *and *7 *after alloxan injection. In some dogs whose plasma glucose levels were below 200 mg/dl at *day 3 *after injection of alloxan, a second injection of alloxan was given. Only dogs with blood glucose >200 mg/dl (fasted for at least 16 h) on *day 7 *were included in the diabetic group. Before and after the development of diabetes, the dogs had free access to water. Systemic hemodynamics were measured again after 3-5 wk. For microarray analysis, DM dogs were sacrificed at 4 weeks after alloxan injection and the LV of the heart was harvested and stored at -80°C for only two weeks before RNA isolation was performed and sent to microarray facility for quality control, labeling and hybridization to Affymetric microarray.

#### Recording Techniques

Arterial pressure, LV pressure, left circumflex coronary blood flow (CBF), and internal diameters were measured; heart rate (HR), mean arterial pressure, and mean CBF were derived in conscious dogs, as we described previously [11]. Measurements were recorded continuously throughout the course of the experiment. Baseline measurements were taken after the dog was resting quietly on the table for 40 min.

### RNA isolation

Total cardiac RNA was extracted at the same time from left ventricular tissue of control (n = 4), and DM dogs (n = 4) with a commercial RNA isolation kit using Trizol (TRI REAGENT, Sigma, Saint Louis Missouri) as described previously [19,20]. RNA samples were stored at -80°C for two weeks before microarray labeling and hybridization. Two quality control measures are carried out on a small aliquot of the isolated RNA samples: 1) a spectrophotometric analysis to confirm the concentration and to detect contaminating proteins and other molecules, and 2) a size fractionation procedure using the Agilent Bioanalyzer 2100 (Agilent Technologies) to determine whether the RNA is intact. The later was performed at the Bionomics Research and Technology Center (BRTC), Rutgers University.

### Microarray labeling and hybridization

The microarray labeling and hybridization procedures were carried out at the Bionomics Research and Technology Center (BRTC), Rutgers University. The microarray labeling, hybridization and analysis procedures were described previously in detail [19]. In brief, each individual sample was subjected to gene expression analysis via the Affymetrix Canine Genome Array, which was subsequently processed and scanned according to the manufacturer's instructions. The GeneChip^® ^Canine Genome Array was the first available commercial canine expression array and contains over 23,813 *Canis familiaris *probe sets to monitor gene expression for over 16,000 *Canis familiaris *full length transcripts.

### Microarray data analysis

All arrays referred to in this study were assessed for "array performance" prior to data analysis. This process involved the statistical analysis of control transcripts that are spiked into the samples themselves and the hybridization cocktail to assess array performance. In addition, several genes have been identified on each array to help assess the overall quality of signal intensity from all arrays. The results of this analysis were helpful to validate the reproducibility of each array at baseline, allowing us to define the lower level of sensitivity sufficient to identify small changes in biologically relevant genes.

Prior to analysis, data from each hybridization were processed using Microarray Suite software, v 5.0 (MAS 5.0) to yield average difference values corresponding to signal intensity for each probe-set. Distinct algorithms were used to determine the absolute call, which distinguishes the presence or absence of a transcript, the differential change in gene expression (increase (I), decrease (D), marginal increase (MI), marginal decrease (MD), and no change (NC)), and the magnitude of change, which is represented as signal log ratio (on a log base 2 scale). In brief, the algorithm which defines the presence or absence of a gene takes into consideration several qualitative and quantitative metrics from the raw data set. T-tests were performed on the normalized signal values prior to exploring additional analyses.

All the hybridization data have been submitted to the National Center for Biotechnology Information (NCBI) Gene Expression Omnibus database GEO: http://www.ncbi.nlm.nih.gov/geo with GEO Accession Numbers for series GSE14887. The raw pixels data were imported into GeneTraffic MULTI and all subsequent analyses were performed on a GeneTraffic server (GeneTraffic v. v. 3.2-11, Iobion Informatics, La Jolla, CA). All the microarray data were analyzed and normalized using a Robust Multi-Chip Analysis algorithm (RMA). The average of all the control animal data sets was used as the baseline for the analysis.

### Statistical Analysis

Hemodynamic data were expressed as mean ± SEM and compared by one-way ANOVA followed by Dunnet's test. For the microarray analysis, statistical significance for changes in gene expression was performed in GeneTraffic using a two-class method (*t*-test) and with variance stabilization. Due to the overwhelming amount of information generated from microarray analysis, differences were considered statistically significant at a nominal significance of *P *≤ 0.01 and at least ± 2 -fold change in expression between control and DM dogs. However, for a subset of genes of interest (such as gp91), we considered *P *≤ 0.05 as a significant value.

## Results

### Hemodynamics and cardiac function

The hemodynamic data for these dogs were measured in this study and they were similar to our previously reported data [11,12,17]. In our previous studies; we have shown that insulin levels in plasma were significantly decreased, whereas glucose levels in plasma were significantly increased during the development of diabetes (all *P *< 0.05). In this current study with new group of animals, we also showed that induction of diabetes mellitus increased arterial glucose levels from 74 ± 2 to 291 ± 32 at *week 1*, to 310 ± 33 at *week 2*, to 317 ± 41 at *week 3*, and to 279 ± 43 at *week 4*, respectively (*P *< 0.05 vs. control) similar to our previous data [17]. Furthermore, dogs had clinical signs of diabetes, i.e., polyuria, polydipsia and weight loss (25.1 ± 0.7 to 20.5 ± 0.9 kg at *week 4*).

In addition, our current data showed that LVSP was reduced (125 ± 6 mmHg to 107 ± 8 mmHg) and LV dP/d*t *did not change from control (2762 ± 216 to 2374 ± 343 mmHg/s) during diabetes. MAP decreased gradually, and this reduction reached statistical significance 102 ± 7 to 91 ± 6 mmHg at *week 4*. Moreover, at *week 4 *of diabetes, heart rate and mean CBF were significantly diminished ( 28 ± 5 ml/min to 18 ± 4 ml/min) and (100 ± 7 to 76 ± 14 beats/min), respectively, compared with control (*P *< 0.05).

### Global gene transcript profiling

**Figure 1:**
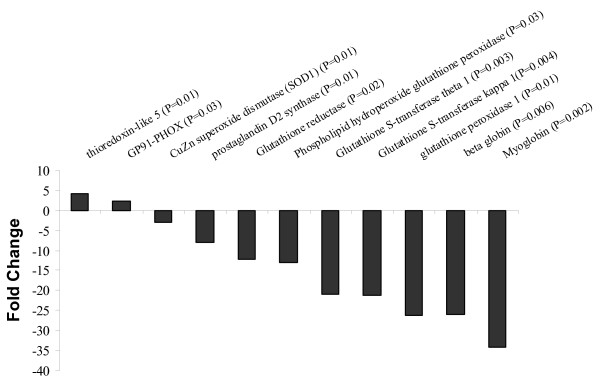
**Fold change of some genes involved in the bioactivity of NO that are statistically differentially regulated in the DM dogs**. P values are shown for each individual gene.

Our microarray analysis was performed on heart tissues taken at 4 weeks after induction of DM. From our current and previously published data [17], this is the time when glucose uptake was low. Earlier times were mainly fueled by keto-acid uptake. When statistical analysis was performed using *P *≤ 0.01 and ± 2.0 fold change in comparison to the control group, our result showed that 797 genes were differentially expressed in DM in comparison to control dogs. Of these, 150 genes were expressed at significantly greater levels in diabetic dogs and 647 were significantly reduced. For a full list of differentially expressed genes see additional file 1, Table S1. Unfortunately, about half of the genes in the dog genome remain unidentified, due to the lack of annotation. When considering the identified genes, we found that the majority of the genes presented encoded for proteins involved in 104 KEGG (Kyoto Encyclopedia of Genes and Genomes) pathways. The top 12 pathways with the highest number of genes involved are shown in Table 1. We have divided some genes into functional categories and have presented examples of some of them in Table 2. These data showed that a large number of the down regulated genes are involved in metabolic pathways (Table 2). There was down regulation of some of the genes involved in the mitochondrial transport system (Table 2). Furthermore, there was differential down regulation of some of the genes involved in Ca^2+ ^cycling and in myocardial contraction and relaxation (Table 2) in addition to down regulation of genes involved in glutathione pathway (Figure 1). There was up regulation of some components of the NADPH oxidase subunits (gp91 by 2.2 fold, P < 0.03) and down-regulation of SOD1 (3 fold, P < 0.001) (Figure 1).

**Table 1:** Shows the top 12 KEEG pathways with the highest number of genes involved.

KEGG Pathway	Number of genes
Ribosome	27
Oxidative phosphorylation	21
Focal adhesion	7
Citrate cycle (TCA cycle)	6
Glutathione metabolism	6
Glycolysis/Gluconeogenesis	6
Regulation of actin cytoskeleton	6
Adherens junction	5
Calcium signaling pathway	5
Leukocyte transendothelial migration	5
PPAR signaling pathway	5
Wnt signaling pathway	5

**Table 2:** Examples of some of the genes which are statistically significant in alloxan induced diabetes in dog hearts

Affy Ids	Fold-change	P Values	Gene Title
**Metabolic genes**			
Up-regulated			
1604393_at	7.31	0.01	inorganic pyrophosphatase
Down-regulated			
1582358_s_at	-78.79	0.004	glyceraldehyde-3-phosphate dehydrogenase
1586136_x_at	-77.17	0.006	cytochrome c oxidase, subunit VIb polypeptide 1
1604455_at	-55.33	0.002	Creatine kinase M-type
1583489_s_at	-54.19	0.009	NADH-ubiquinone oxidoreductase 23 kDa subunit, mitochondrial precursor (Complex I-23KD)
1589664_s_at	-26.35	0.002	ATP synthase, H+ transporting, mitochondrial F0 complex, subunit c, isoform 1
1603350_x_at	-25.11	0.003	Beta enolase (2-phospho-D-glycerate hydro-lyase)
1603755_at	-24.59	0.005	NADH dehydrogenase (ubiquinone) Fe-S protein 6, 13kDa (NADH-coenzyme Q reductase)
1586831_at	-24.42	0.006	Phosphoglycerate mutase 2
1583253_at	-22.94	0.006	NADH-ubiquinone oxidoreductase Fe-S protein 7
1604453_at	-22.63	0.003	isocitrate dehydrogenase 2 (NADP+), mitochondrial
1594799_at	-22.47	0.007	NADH dehydrogenase (ubiquinone) Fe-S protein 3, 30kDa (NADH-coenzyme Q reductase)
1583615_at	-20.11	0.01	inositol polyphosphate-5-phosphatase, 40kDa
1588757_at	-17.75	0.003	NADH dehydrogenase (ubiquinone) 1 beta subcomplex, 7
1597725_s_at	-17.63	0.006	3-hydroxyacyl-CoA dehydrogenase type II
1583148_at	-17.39	0.008	ubiquinol-cytochrome c reductase core protein I
1583152_at	-17.27	0.007	NADH dehydrogenase (ubiquinone) 1 alpha subcomplex, 8, 19kDa
1583193_s_at	-15.89	0.003	isocitrate dehydrogenase 3, beta subunit isoform b precursor
1592397_at	-15.24	0.009	succinate-CoA ligase, GDP-forming, alpha subunit
1588432_at	-14.22	0.009	cytochrome c-1
1588302_at	-13.55	0.004	ubiquinol-cytochrome c reductase subunit
1583550_s_at	-13.55	0.008	Vacuolar ATP synthase 16 kDa proteolipid subunit
1585498_at	-12.82	0.01	Glucose-6-phosphate isomerase (GPI)
1583430_at	-12.64	0.01	Triosephosphate isomerase (TIM) (Triose-phosphate isomerase)
1585763_at	-12.64	0.008	Ubiquinol-cytochrome c reductase complex 11 kDa protein, mitochondrial precursor
1583348_x_at	-12.55	0.006	cytochrome c oxidase, subunit VIb polypeptide 1
1604126_at	-10.63	0.007	enoyl Coenzyme A hydratase, short chain, 1, mitochondrial
1584695_at	-7.84	0.003	succinate dehydrogenase complex, subunit B, iron sulfur (Ip)
1604230_at	-7.73	0.006	NADH dehydrogenase 1 beta subcomplex 4
1598523_at	-6.02	0.01	Glucose-6-phosphate isomerase (GPI)
1589790_s_at	-6.02	0.007	NADH dehydrogenase (ubiquinone) 1 alpha subcomplex, 6, 14kDa
1592755_s_at	-5.24	0.008	Electron transfer flavoprotein-ubiquinone oxidoreductase
1588377_at	-5.17	0.01	3-methyl-2-oxobutanoate dehydrogenase kinase
1598623_s_at	-4.79	0.005	phosphofructokinase, muscle
1586218_at	-4.76	0.004	Aldose reductase
1590058_s_at	-4.56	0.002	creatine kinase, mitochondrial 2
1584362_at	-4.44	0.01	NAD-dependent deacetylase sirtuin-3
1583716_at	-4.03	0.004	aconitase 2, mitochondrial
			
**Mitochondria proteins and transport**			
**Down-regulated**			
1583895_x_at	-71.51	0.008	ribosomal protein L29
1593894_at	-22.78	0.01	Mitochondrial import receptor subunit TOM22 homolog
1587993_x_at	-11.47	0.008	Inner membrane protein OXA1L, mitochondrial precursor (Oxidase assembly 1-like protein)
1585706_x_at	-11.39	0.008	Inner membrane protein OXA1L, mitochondrial precursor (Oxidase assembly 1-like protein)
1588228_at	-9.85	0.006	Mitochondrial 39 S ribosomal protein L23 (L23mt) (MRP-L23) (L23 mitochondrial-related protein)
1592554_at	-9.06	0.004	14-3-3 protein epsilon (14-3-3E) (Mitochondrial import stimulation factor L subunit) (MSF L)
1586690_at	-7.89	0.01	NTF2-related export protein 1
1585137_at	-7.52	0.01	Mitochondrial 2-oxoglutarate/malate carrier protein (OGCP)
1586284_at	-7.52	0.009	ATP synthase coupling factor 6, mitochondrial precursor (ATPase subunit F6)
1582738_at	-7.26	0.01	FXYD domain containing ion transport regulator 1 (phospholemman)
1586934_at	-7.11	0.008	Solute carrier family 2, facilitated glucose transporter, member 1 (Glucose transporter type 1)
1592219_s_at	-4.44	0.01	transaldolase 1
			
**Calcium uptake and myocardial contraction and relaxation**			
Up-regulated			
1606083_at	4.44	0.007	protein phosphatase 3 (formerly 2B), catalytic subunit, alpha isoform (calcineurin A alpha)
Down-regulated			
1582664_s_at	-48.50	0.004	troponin I type 3 (cardiac)
1582739_s_at	-36.50	0.01	FXYD domain containing ion transport regulator 1 (phospholemman)
1586044_s_at	-20.82	0.008	actinin, alpha 2
1585703_s_at	-19.97	0.006	beta-actin///actin, gamma 1
1582670_s_at	-16.56	0.006	myosin, light chain 2, regulatory, cardiac, slow
1592467_at	-15.03	0.01	Tropomyosin 1 (alpha)
1605421_at	-13.27	0.009	phosphodiesterase 1C, calmodulin-dependent 70kDa
1587427_at	-11.24	0.008	ryanodine receptor 2 (cardiac)
1605889_at	-9.85	0.004	Myosin, light polypeptide 2, regulatory, cardiac, slow
1605583_at	-5.17	0.009	cardiac titin
1585044_x_at	5.13	0005	myosin, light polypeptide 6, alkali, smooth muscle and non-muscle
1589676_s_at	-5.06	0.006	actin, alpha, cardiac muscle 1
1583557_at	-4.86	0.008	Adenylyl cyclase-associated protein 1 (CAP 1)
1584317_at	-4.86	0.005	smoothelin isoform c
1602057_s_at	-4.50	0.01	calmodulin 1
1604456_at	-4.44	0.004	troponin C, slow
1592238_s_at	-2.40	0.01	SERCA2

## Discussion

Some of the genes that we found markedly up- or down regulated encode for proteins whose role in myocardial physiology and pathophysiology is well defined. Other genes are presently difficult to put in a pathophysiolgical context. It is obviously impossible to examine in detail functions and potential pathophysiological roles of all of the identified alterations, therefore our discussion will focus on selected genes whose expression was found markedly altered and whose potential importance is supported by the existing literature.

### Regulation of cardiac metabolic and structural genes

A large number of the down regulated genes are involved in metabolic pathways. Interestingly, as seen in Table 2, the majority of the genes in the citric acid cycle and the respiratory chain are down regulated by many fold decrease. The large down regulation of these genes may be indicative of mitochondrial dysfunction in DM dogs. In our previous studies [12], we have shown that there is depressed modulation of O_2 _consumption by endogenous NO in cardiac muscle from diabetic dogs. However, in this recent study based on our gene expression profiling of cardiac tissues from DM dogs, there is strong evidence of a dysfunction of the mitochondria in this DM model. This was also evident by the down regulation of some of the genes involved in the mitochondrial transport system. Examples of these are shown in Table 2. Of particular interest are mitochondrial import receptor subunit TOM22, mitochondrial import stimulation factor L subunit, mitochondrial 2-oxoglutarate/malate carrier protein, glucose transporter type 1, voltage-dependent anion-selective channel protein 3, malonyl CoA-acyl carrier protein transacylase, translocase of inner mitochondrial membrane 50 homolog. This conclusion supports previous observations by other investigators that there is evidence for mitochondrial dysfunction in the hearts from other models of diabetes [13-15,21]. Savabi [21] showed that there were fewer mitochondria in the hearts from diabetic rats and lower O_2 _consumption rates in isolated mitochondria than those from normal hearts. Other studies [16] also suggested that there was depressed basal O_2 _consumption by myocytes suspended in medium, decreased calcium uptake by mitochondria, and reduced mitochondrial membrane potential from diabetic rats. The decrease in calcium uptake by mitochondria is also supported in this study in dogs with DM. Table 2 shows examples of some of the genes involved in Ca^2+ ^cycling and in myocardial contraction and relaxation. During the last decade there was accumulating evidence that alterations of excitation-contraction coupling (EC) may play a critical role in the pathophysiology of myocardial failure. Based on our microarray data (Table 2), a large number of genes involved in these processes are down regulated. The level of mRNA of troponin I, troponin C, tropomyosin, calmodulin, ryanodine receptor type 2 (RyR) and SERCA2 were significantly decreased in these animals. Troponin I has been shown to be very sensitive and specific indicator of damage to the myocardium. Another gene which was down regulated was phospholemman which is a cardiac transmembrane protein. Phospholemman has been shown to regulate ion channel activity [22]. It has been proposed that phospholemman regulates the Na/K pump in a manner analogous to regulation of the calcium ATPase SERCA 2a by phospholamban [22]. The net effect of these changes in gene expression might be an overall decrease in both diastolic and systolic ventricular function, which may be an adaptive mechanism to protect the surviving myocardium by reducing its energy expenditure. Linke *et al *[17] and Zhao *et al *[11,12] showed that in alloxan-induced diabetes in dogs there were no changes in LVSP and LV dP/dt at rest. In view of the role of sarco/endoplasic reticulum in the cardiac contraction and relaxation process, the observed reduction in gene expression of sarco/endoplasic reticulum calcium handling proteins may explain the depressed cardiac performance in diabetic dogs. Although our sampling protocol focused on the myocardium, others have also shown similar observations in vascular smooth muscle cells (VSMCs) [23]. Searls *et al s*howed that the previously-reported reduced Ca^2+ ^signaling in VSMCs from diabetic animals is likely to be due to the altered distribution and/or levels of Ca^2+ ^regulatory proteins including the IP_3_R Ca^2+ ^channels, RyR and SERCA proteins [23].

### Regulation of oxidative stress genes

A number of studies suggested that superoxide is involved in the endothelial dysfunction in experimental diabetic animals and patients with diabetes because superoxide dismutase and other antioxidants could, at least in part, restore the endothelial dysfunction. We have previously shown that in conscious dogs with DM that: *1*) baseline CBF was significantly decreased, *2*) endothelium-dependent (ACh) coronary vasodilation were impaired, and *3*) reflex cholinergic NO-dependent coronary vasodilation was selectively depressed. The most likely mechanism responsible for the depressed reflex cholinergic NO-dependent coronary vasodilation was the decreased bioactivity of NO from the vascular endothelium. Bradykinin reduced oxygen consumption in normal dog heart in vitro and this was reduced in type I DM [12]. The effect of bradykinin was restored by apocynin, tempol or vitamin C; indicating a role for NADPH oxidase derived superoxide. Interstingly, there was up regulation of some components of the NADPH oxidase subunits (gp91 by 2.2 fold, P < 0.03) and down-regulation of SOD1 (3 fold, P < 0.001). Over the years, decreased NO bioavailability has been proposed as one of the determinants of vascular damage in diabetes. Recently, Hamed *et al *have shown that NO and superoxide dismutase play roles in modulating endothelial progenitor cell function in type 2 diabetes mellitus [24].

Oxidative stress is involved in the pathogenesis of diabetic complications in animal models and in humans [3]. Many authors believe that the liver mitochondrial dysfunction in diabetes is closely dependent on oxidative stress which is enhanced in diabetic animals and patients [25,26]. Long-term reactive oxygen species exposure resulted in damage of mitochondrial proteins that caused disturbances in mitochondrial energy production [27]. Some authors suggested that the loss of mitochondrial respiration was essentially due to oxygen-dependent inactivation of mitochondrial dehydrogenases [28]. The mitochondrial dehydrogenase activities related to complex I (NADH-dehydrogenase) and to complex II (succinate dehydrogenase) were shown to decrease in the diabetic rats. Interestingly, all of the above mentioned genes are also down regulated in our model presented in this study (Table 2).

Oxidative stress may be defined as an imbalance between the production and degradation of reactive oxygen species such as superoxide anion, hydrogen peroxide, lipid peroxides, and peroxynitrite. Enzymatic inactivation of reactive oxygen species is achieved mainly by glutathione peroxidase, superoxide dismutase, and catalase [29]. The peroxidation of membrane lipids is a major consequence of oxidative stress. The oxidation of lipids, particularly phospholipids, has also been suggested to be a significant chemical event in a variety of pathological conditions, such as atherosclerosis, ischemic injury, and carcinogenesis [30]. The formation of lipid hydroperoxides (LOOHs) within the bilayer of membranes disrupts membrane structure and function [31], which subsequently leads to cell injury and death. The repair of intracellular LOOHs can be carried out by cytosolic glutathione peroxidase (cGPx) [32], phospholipid hydroperoxide glutathione peroxidase (PhGPx) [33], nonselenium GPx [34], or glutathione-S-transferase (GST) [35].

In mammalian cells, glutathione and the glutathione peroxidases constitute the principal antioxidant defense system [36]. This is evident by the down regulation of phospholipid hydroperoxide glutathione peroxidase, glutathione peroxidase 1, glutathione reductase (mitochondrial precursor) and glutathione S-transferase. These genes were down regulated by 3.7, 4.7, 3.5 and 2.9 fold respectively (Figure 1). There are at least four different glutathione peroxidases, all of which contain selenocysteine at their active sites [37]. Glutathione peroxidase 1, the ubiquitous intracellular form and key antioxidant enzyme within most cells, including the endothelium, uses glutathione to reduce hydrogen peroxide to water and lipid peroxides to their respective alcohols, and it also acts as a peroxynitrite reductase [38]. In mice, glutathione peroxidase 1 deficiency results in abnormal vascular and cardiac function and structure [39]. Recent studies in human have shown that decreased GPX-1 activity was observed in patients with coronary artery disease (CAD) and those with acute myocardial infarction [40]. Espinola-Klein *et al *[41] have also shown that decreased red blood cell GPX-1 activity is associated with increased cardiovascular risk according to the extent of atherosclerosis.

Glutathione is important in the regulation of the redox state, and a decline in its tissue level has often been considered to be indicative of increased oxidative stress in diabetes. Glutathione (GSH) is present in most mammalian cells and plays an important role in cellular defense against oxidative stress by reducing protein disulfides and other cellular molecules. It also acts as a scavenger of free radicals of ROS [42]. GSH is synthesized intracellularly by two GSH-synthesizing enzymes, g-glutamylcysteine synthetase (g-GCS) and glutathione synthetase. g-GCS catalyzes the rate-limiting step of GSH synthesis [43]. In many cells, the GSH redox cycle is catalyzed by both glutathione peroxidase and glutathione reductase. During oxidative stress, the reduced form of GSH is converted by glutathione peroxidase to oxidized glutathione (GSSG). It was reported the presence of low GSH and high GSSG concentrations in erythrocytes of diabetic patients [44] and in endothelial cells of diabetic rabbits [16].

Two of the genes that were highly down-regulated in DM were Myoglobin (Mb) and beta globin (Figure 1). Previous studies have shown the role of myoglobin in the antioxidant defense of the heart [45]. The lack of myoglobin has been shown to cause a biochemical shift in cardiac substrate utilization from fatty acid to glucose oxidation [46]. In our previous studies [17] we have shown that there was a shift in metabolic uptake by the heart during development of alloxan-induced diabetes at a time when myocardial NO production is already decreased (3-4 weeks). This switch is a well know marker for heart failure. Studies have also suggested that human Mb actively participates in the regulation of NO [47]. Mb is a scavenger of bioactive NO through the reaction NO with MbO_2 _to form metMb and nitrate and may be the major mechanism of attenuating intracellular NO bioactivity in cardiac muscle [45]. Flogel *et al *showed that hearts from Mb-deficient (myo-/-) mice were found to be more sensitive to the infusion of reactive oxygen species (ROS, e.g., H_2_O_2 _and superoxide) in that depression of myocardial contractile force was more pronounced as compared with WT controls. Furthermore, Mb-deficient hearts released significantly more ROS during IR, and this was accompanied by a delayed functional and metabolic recovery after the ischemic insult. It is obvious in this study that the array data show a direct effect of diabetes on the heart by impairing NO bioavailability through oxidative stress. Therefore, our study is novel in that it represents the first report of cardiac gene expression changes in a canine model of alloxan-induced type 1 diabetes.

### Limitations

Although the analysis of gene expression using oligonucleotide microarrays is a powerful technique, limitations warrant mention. Not all dog genes are represented on the Affymetrix GeneChip^® ^Canine Genome Arrays used in this study. Furthermore, the annotations of high number of the genes are still unknown and therefore the knowledge that can be acquired from these experiments remains incomplete. Up to now, this version of the Canine array contains 7,340 complete annotated dog genes. From our previous publications [19,20,48,49] in addition to other reported publication [50], there seems to exist a good correlation between oligonucleotide microarray and qRT-PCR data. This correlation also holds true when ratios of gene expression in different tissues were compared for highly expressed genes and not for those expressed at low levels due to the smaller dynamic range of microarrays [50]. It is well known that alloxan is specifically cytotoxic to the pancreatic ß cells. However, the precise mechanism for this selectivity is still not clear. Several studies in alloxan metabolism [51] have documented that 45% of injected alloxan had been excreted in the urine in 24 hours and 62% in 3 days. Therefore, the gene expression data from this study where dogs were sacrificed after 4 weeks of alloxan injection are most likely due to the chronic effect of diabetes rather than the acute toxic effect of alloxan.

## Conclusion

The gene expression profiling presented in this study suggests a direct effect of type 1 diabetes on the heart by impairing NO bioavailability through oxidative stress, impairing calcium uptake by the mitochondrial transport system, lipid peroxidases and down regulation of citric acid cycle and the respiratory chain which are indicative of mitochondrial dysfunction in DM dogs. The order of these events is hard to elucidate and is yet to be determined.

## List of abbreviations

DM: diabetes mellitus; CBF: coronary blood flow; NO: nitric oxide; eNOS: endothelial nitric oxide synthase; SOD: superoxide dismutase; HF: heart failure; O_2_: oxygen; LV: left ventricle; LVSP: LV systolic pressure; HR: heart rate; MAP: mean arterial pressure.

## Competing interests

The authors declare that they have no competing interests.

## Authors' contributions

CO designed the gene array study, performed all the microarray and data analysis and wrote the manuscript. SK Performed the hemodynamics on the animals. FAR participated in editing the manuscript and THH designed the study and revised and approved the final manuscript. All authors have read and approved the manuscript.

## Supplementary Material

Additional file 1 **Table S1**. Full list of genes with increased and decreased expression in DM versus control dogs. Click here for file
